# Controlling Population Evolution in the Laboratory to Evaluate Methods of Historical Inference

**DOI:** 10.1371/journal.pone.0002960

**Published:** 2008-08-13

**Authors:** Patrick Mardulyn, Marie-Anne Vaesen, Michel C. Milinkovitch

**Affiliations:** Laboratory of Evolutionary Genetics, Université Libre de Bruxelles, Gosselies, Belgium; Indiana University, United States of America

## Abstract

Natural populations of known detailed past demographic history are extremely valuable to evaluate methods of historical inference, yet are extremely rare. As an alternative approach, we have generated multiple replicate microsatellite data sets from laboratory-cultured populations of a gonochoric free-living nematode, *Caenorhabditis remanei*, that were constrained to pre-defined demographic histories featuring different levels of migration among populations or bottleneck events of different magnitudes. These data sets were then used to evaluate the performances of two recently developed population genetics methods, BayesAss+, that estimates recent migration rates among populations, and Bottleneck, that detects the occurrence of recent bottlenecks. Migration rates inferred by BayesAss+ were generally over-estimates, although these were often included within the confidence interval. Analyses of data sets simulated *in-silico*, using a model mimicking the laboratory experiments, produced less biased estimates of the migration rates, and showed increased efficiency of the program when the number of loci and sampled genotypes per population was higher. In the replicates for which the pre-bottleneck laboratory-cultured populations did not significantly depart from a mutation/drift equilibrium, an important assumption of the program Bottleneck, only a portion of the bottleneck events were detected. This result was confirmed by *in-silico* simulations mirroring the laboratory bottleneck experiments. More generally, our study demonstrates the feasibility, and highlights some of the limits, of the approach that consists in generating molecular genetic data sets by controlling the evolution of laboratory-reared nematode populations, for the purpose of validating methods inferring population history.

## Introduction

Significant advances in theoretical population genetics, as well as the increasing facility with which molecular genetic data are produced, have motivated the recent development of new analytical methods for estimating, from molecular data, population evolution parameters [1]–[3] such as the effective size of a population and its fluctuations over time [4]–[6], the migration rates among sub-populations [7], [8], or the divergence time between two isolated populations [9]. These methods are often implemented in computer programs that are freely available (reviewed in [2]) and provide potentially powerful tools to study the past demography of populations.

One fundamental problem with methods of historical inference is that their performances cannot be easily evaluated. Computer simulations, *i.e.*, implementing *in silico* evolution of virtual population(s), allow identifying conditions under which a method exhibits low statistical power and/or various types of biases (e.g., [10]–[12]). The interest of computer simulations consists in the ability to rapidly generate a large number of replicate data sets, under various historical scenarios, therefore allowing the exploration of a wide range of parameters. These approaches may however present some weaknesses: models (*e.g.*, the nucleotide substitution model or the coalescent model of population evolution) under which the evolution of virtual populations are simulated and the model used for performing the inference generally share a number of simplifying, untested, assumptions about the true evolutionary processes. Thus, while simulation studies are essential to identify conditions under which a method can fail to properly estimate a given parameter, we may not be able to entirely rely upon them to fully validate a method of historical inference.

In order to deal with the potential problems associated with model over-simplification, one would ideally complement simulation studies with the analysis of a series of real populations of known history. However, natural populations of known detailed past demography are extremely rare. Hence, an intermediate potentially useful approach may consist in using populations of laboratory-reared organisms that are constrained to follow a pre-defined demographic scenario. It offers two advantages over the study of natural populations: *(i)* different parameters of the population evolution can be precisely defined by the experimenter and *(ii)* multiple replicates of each demographic scenario can be performed. This approach avoids making many (but not all) of the simplifying and untested assumptions that are necessary in numerical simulations. Populations of laboratory-reared organisms have already been used in the past for similar purposes: *e.g.*, laboratory *Drosophila* populations to study the effect of inbreeding/drift on genetic diversity in small populations [13], [14], or T7 bacteriophage populations to evaluate a statistical method distinguishing between the effect of selection and population expansion on genetic variation [15]. Similarly, to validate several commonly-used methods of molecular phylogeny inference, Hillis et al. [16] have used data from serially propagated T7 bacteriophage populations in the laboratory in the presence of a mutagen and with an imposed specific pre-defined phylogeny.

Here, we explore the possibility of developing a similar experimental approach by generating multiple replicate microsatellite data sets from laboratory-cultured populations of a gonochoric free-living nematode, *Caenorhabditis remanei* (closely related to the hermaphrodite species *C. elegans*, [17]), that were constrained to pre-defined demographic histories featuring different levels of migration among populations or bottleneck events of different magnitudes. *C. remanei* was chosen because it is characterized by a very short generation time (3–4 days), allowing a relatively high number of generations in a reasonably low amount of time. Previous studies [18], [19] have found evidence of high nucleotide variation in *C. remanei* natural populations, about 20-fold higher than in the closely related self-fertilizing species *C. elegans* and *C. briggsae*. We suggest that data sets generated in this way could serve as preliminary models for testing current and future analytical methods whose objectives are to estimate migration rates among populations or to detect past bottleneck events. We also suggest that controlling demographic history in laboratory-cultured populations could be extended to test and validate historical inference methods under a wide range of realistic parameters. We used our *C. remanei* generated data sets to evaluate the performances of two recently developed population genetics methods, implemented in the programs BayessAss+ [7], that estimates recent migration rates among populations, and Bottleneck [20], that detects the occurrence of recent bottlenecks. *In-silico* simulated data were also generated under conditions similar to the conducted laboratory experiments, to compare the behavior of BayesAss+ with *in-silico* and *in-vitro* simulated data sets.

## Materials and Methods

### Laboratory cultures of C. remanei

Several outbred strains of *C. remanei*, originally collected from different parts of the world, were obtained from the Caenorhabditis Genetics Center (http://www.cbs.umn.edu/CGC/). Nematodes were cultured at 20°C in 10 ml of a liquid medium (described in [21]) containing 20 mg/L of the antibiotic nalidixic acid and inoculated with the nalidixic-acid-resistant XA106F^−^ strain of *E. coli*. Culture flasks were continuously stirred at 100 rpm. Homogenization was however interrupted 2 hours per day to allow for sexual reproduction. Half of the homogenized liquid culture was replaced by fresh medium once per week. Populations involved in the migration experiments were from different strains of *C. remanei* (PB206, collected in Dayton, Ohio, USA by S. Baird; EM464, collected in Brooklyn New York, USA by S. Emmons; SB146, collected in Freiburg, Germany by B. Wood). In bottleneck experiments, populations were generated through mixing 7 different strains (PB206, EM464, SB146, PB219, PB212, PB228, PB229) for increasing allelic diversity.

### Experimental design

In all experiments, one 10 ml culture flask was considered a separate population. Each experiment described below was conducted in five replicates.

**Figure 1:**
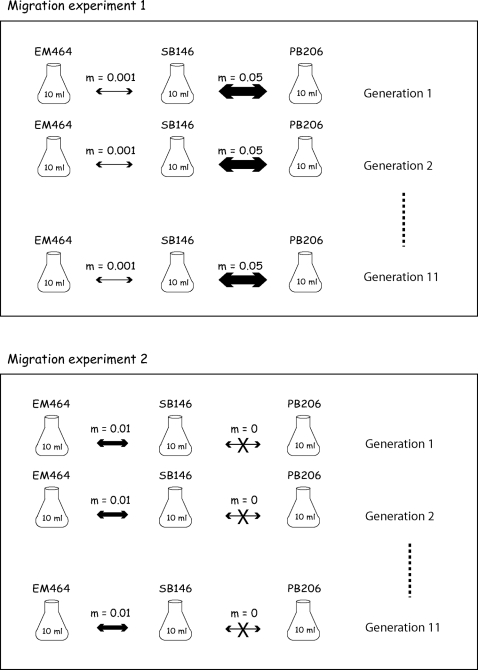
Experimental designs used to evaluate the inference of recent migration rates. Each population is represented by a liquid-culture flask. A double arrow separating two flasks shows the migration rate that was implemented at each generation. The name of the *C. remanei* strain is indicated above each population. Each experiment was run in 5 replicates.

**Figure 2:**
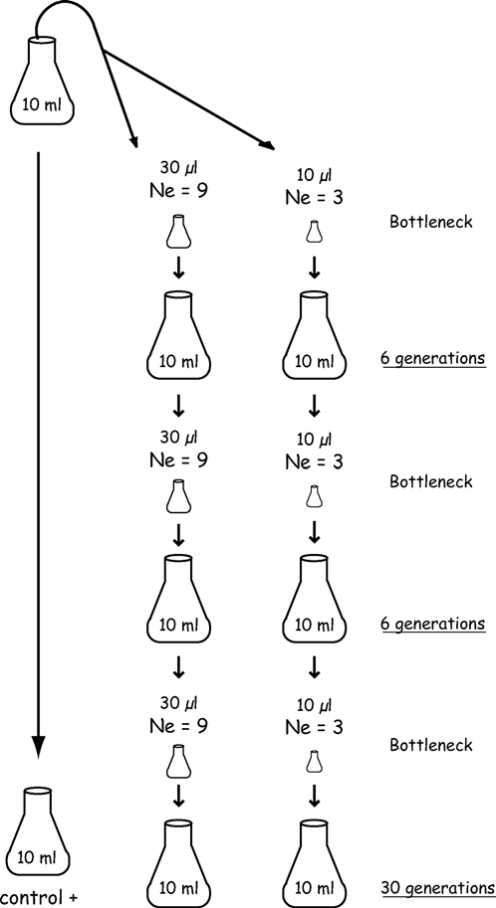
Experimental design used to evaluate the detection of a bottleneck event. Each population is represented by a liquid-culture flask. At the beginning of the experiment, a source population was used to create two new populations from two different small numbers of founder individuals (corresponding to N_e_ = 3, and N_e_ = 9). These bottlenecked populations were submitted to two additional bottleneck events of the same magnitude. The source population is used as the control population (i.e., experiencing no bottleneck). This experiment was run in 5 replicates.

a) Migration experiments (Figure 1): two different experiments were designed that both included three populations among which different levels of migration were implemented. Migration events were performed twice per week (assuming a generation time of 3.5 days for laboratory cultured populations, [22]) by transferring the appropriate homogenized volume of liquid culture from one population to the other with a pipettor, in one direction, then in the reverse direction (gene flow between each population pair was always symmetrical). Note that following this procedure, some of the migrants transferred in one direction are taken back to the first population when implementing the reverse migration. The real migration rates are therefore slightly smaller than the ones announced in Figure 1 (0.0475, 0.0099, and 0.000999 instead of 0.05, 0.01, and 0.001, respectively), but these differences are negligible when compared to the confidence intervals estimated from the genetic data (see results). In both experiments, migration was conducted during 11 generations, after which samples were collected from each population (24 nematode individuals) for genotyping.

b) Bottleneck experiments (Figure 2): A source population (different for each replicate) was generated by mixing seven strains of *C. remanei* (see above) and maintained for about 100 generations before starting the experiment. This was done to artificially increase the level of genetic polymorphism, as preliminary tests indicated that each strain taken separately was characterized by an insufficient level of polymorphism. Then, two new populations were created from this source population by transferring a small volume (30 µl and 10 µl) of it into a new 10 ml culture flask. Two extra bottleneck events of the same magnitude were implemented in these populations each time after waiting six generations to allow the population to recover a reasonable size (as checked by estimating the census size–see below). The source population was kept as the control population and was never subject to a bottleneck event. After the last bottleneck event, the cultures were maintained for 30 generations before sampling of 32 individuals per population.

### Isolation of polymorphic microsatellite loci

A genomic library was constructed and then enriched for microsatellite loci following a protocol similar to the one described by Glenn and Schable [23]. Briefly, genomic DNA was extracted from a 1-liter liquid culture of *C. remanei* using a standard phenol/chloroform extraction protocol [24]. Genomic DNA was restricted with Sau3AI. Restricted fragments were ligated on both ends to a double-stranded linker that was used later as primer-binding sites for PCR amplification. The ligation product was migrated on a 1% agarose gel and fragments of size 800–1,200bp were extracted and purified with the Qiaquick Gel Extraction Kit (Qiagen). In a series of trial and error experiments, hybridization of fragmented and denatured genomic DNA (gDNA) with different microsatellite biotinylated oligonucleotide probes was conducted at different temperatures in the presence of streptavidin-coated magnetic beads. The hybrids gDNA/oligonucleotide probes were isolated using a magnet and the hybridized gDNA was recovered through a series of washing steps. The product of this procedure was PCR amplified (using primers binding to the previously added linkers) with the Long Expand Template PCR System Kit (Roche), and PCR products were ligated to the *pSTC1.3* no-background vector (*StabyCloning™* kit, Delphi Genetics) and transformed into competent cells (Delphi Genetics). Recombinant molecules were isolated from clones and sequences of inserted genomic DNA fragments were obtained by cycle sequencing followed by electrophoresis on an ABI 3730 sequencer (Applied Biosystems). Hybridization with a (GA)_14_ probe at 65°C provided the highest proportion of microsatellite loci for this species. From all isolated loci, 26 were selected on the basis of their length (10–25 repetitions) and tested for polymorphism by genotyping several individuals from different strains. Finally, 10 loci with enough variability and unambiguous amplification patterns were selected for genotyping nematodes in our migration and bottleneck *in-vitro* simulations.

### Estimation of population size

The census size (N) of each population was estimated at different times by counting the number of individuals present in 5 samples of 10 µl of homogenized liquid culture under a dissecting microscope. The effective population size (N_e_) was estimated once for 2 of the source populations used for the bottleneck experiments. This was done by genotyping two samples of 32 individuals in the same population at 4 generations interval. N_e_ was estimated using the program MN_e_ [6], that allows the estimation of N_e_ from genotype data collected at different time points.

### Genotyping of sampled individuals

A quick and easy method was used to extract DNA from single nematodes. Each individual was placed in a 0.2 ml microtube containing 10 µl of a lysis buffer (Tris-HCl 10 mM pH 8.2, KCl 50 mM, MgCl_2_ 2.5 mM, Tween-20 0.45%, gelatin 0.01%, proteinase K 60 µg/ml). Each microtube was then incubated at −80°C for 30 minutes, at 65°C for 1 hour, and finally at 95°C for 15 minutes. Microsatellite loci were amplified by PCR using the following primers and annealing temperatures: locus 1 (GTTTCTTTCTTTTTGCTCTCTTGCTCC, CTCCTGCTCTTGCCTCCC, 58°C), locus 7 (GTTTCTTAGACCTACCCCTACCTGCT, AGCCCAATTCCCCACCTTTT, 60°C), locus 10 (GTTTCTTCTTCGTTGTCTTCCTTCTTC, CCCTCCACCCGACCTTC, 58°C), locus 12 (GTTTCTTGAGACGAAAATAGAGAGAAA, AGAGAAGAGAAATAGAGAAT, 52°C), locus 16 (GTTTCTTTCGTTCATCTTTTTCTTCAT, GGGGGTACCTTTGAATAG, 52°C), locus 22 (GTTTCTTCCATGACTACCACCCAAACA, CGGATCCACAATTTCACTTC, 58°C), locus 28 (GTTTCTTCCCTGCCAAATTATACCAAC, TTCCCTTTTCTCTGCGTCT, 54°C), locus 33 (GTTTCTTAAGAGGGAAGAAAGTGACGAGAA, GTTGTAGTTGTTGTTGTCGTAGTTG, 64°C), locus 36 (GTTTCTTGCATCCGTCATATTCTT, TTTCTTCTCCGTTCTCT, 48°C), locus 37 (GTTTCTTTCCTCGTCGAGTTGTTTATAC, GGTGTTGATATAGCTGCCGAG, 63°C). The PCR conditions were as follows: after an initial denaturation step of 4 min 30 sec at 95°C (activation of FastStart Taq from Roche); 40 cycles of 1 min at 95°C, 2 min at the annealing temperature (see above), and 2 min at 72°C; followed by a final elongation step of 60 min at 72°C. Amplified products were separated by electrophoresis on an Applied Biosystems 3730 automated sequencer. Allele call was conducted with the software GeneMapper version 3.7 (Applied Biosystems). A few of the genotypes appeared to display three, or even four alleles. We believe this is due either to the erroneous sampling of more than one individual per tube during DNA extraction or to the presence of fertilized eggs (which therefore may have received different alleles from the father) extracted along with female individuals. We have dealt with this problem in two ways. First, when the peak height of one allele was clearly smaller than the others (i.e., max 20% of the allele with highest amplitude), the corresponding allele was considered not belonging to the main genotyped individual. Second, when peak heights of all alleles were similar, and when more than two alleles were found for one locus, the corresponding individual was deleted from the data set. Often, the presence of more than two alleles at one locus was detected for several loci, allowing us to clearly identify problematic individuals that could subsequently be discarded from the data set.

### Genetic variation

Gene diversity (sensu Nei [25], i.e., expected heterozygosity) was calculated for all populations. F_ST_ values were estimated, as in Weir and Cockerham [26], among pairs of populations for the laboratory migration experiment data using the program GenePop [27], version 3.4. The Garza-Williamson index (the number of alleles divided by the allelic range) [28], expected to be low in bottlenecked populations, was measured for all populations of the Bottleneck experiment using Arlequin [29], version 3.1.

### Estimating migration rates from the microsatellite data

The multilocus genotype data sets obtained from our migration experiments were analyzed with BayessAss+ version 1.3 to estimate the migration rates between pairs of populations. Each data set was analyzed 5 times (10 million iterations, burn-in of 999,999 iterations), and associated likelihood values were subsequently compared among runs. For comparison, long-term migration rates were also estimated for one of the replicate of the first migration experiment (Figure 1, above), in two different ways. First, migration rates were directly inferred from the F_ST_ values calculated between population pairs, assuming an island model of migration [30], with the derived formula F_ST_ = 1/(4Nm+1) (e.g.,[31]). Second, long-term migration rates were inferred through maximum likelihood, with a coalescent-based model, using the program Lamarc [32] version 2.1.2 (for each estimate, 3 independent runs were compared, each with 10 initial chains (500 trees sampled) and 2 final chains (10,000 trees sampled), burnin = 1,000; confidence intervals based on percentile profiling). In both cases, the effective size was set to 3,000 to infer the migration rate m.

### Detecting bottleneck events from the microsatellite data

For each generated data set, using the program Bottleneck version 1.2.02, we performed a Wilcoxon sign-rank test to determine whether a significant number of loci featured a heterozygosity excess, which is indicative of a recent botteneck event, assuming a two-phase mutation model (TPM; model of microsatellite mutation; [20]).

### In-silico Simulations

We also simulated *in-silico* the evolution of populations under conditions similar to those experienced by the laboratory populations, to compare the behavior of both programs with *in-silico* and *in-vitro* simulated data sets. For the migration experiments, populations with an effective size of 3,000 individuals were simulated, exchanging migrants at the same rate as in the laboratory experiments. Each *in-silico* simulated population was initiated from 100 randomly picked genotypes observed in the single strain populations involved in the migration laboratory experiment. These 100 initial genotypes were then used to generate *in-silico*, through simulated sexual reproduction, a second-generation population of 3,000 individuals (i.e., the estimated effective populations size in the laboratory experiments). The *in-silico* populations were then constrained to a constant population size of 3,000 and experienced random sexual reproduction for 100 generations with no migration and no mutation. Inheritance was considered fully independent among loci. Then, migration was simulated for 11 generations following the scheme described in Figure 1. At the last generation, just before sampling, reproduction of parent individuals generated 81000 individuals (i.e., the mean census size of the real populations). Then, 20, 50, and 100 virtual individuals were sampled and their genotypes recorded. Each *in-silico* simulation was conducted five times, resulting in an equal number of replicates than for the *in-vitro* laboratory simulations. Analyses of these *in-silico* generated data were performed with BayesAss+ exactly as described for the laboratory-generated data.

For the bottleneck experiments, populations with an effective size of 3,000 individuals were simulated, and experienced a bottleneck of the same magnitude as in the *in-vitro* experiments. Each *in-silico* simulated population was initiated from 150 randomly picked genotypes observed in the mixed-strains populations generated for the bottleneck laboratory experiment (genotypes recorded before implementing the bottleneck events in the laboratory experiment). These 150 initial genotypes were then used to generate *in-silico*, through simulated sexual reproduction, a second-generation population of 3,000 individuals (i.e., the estimated effective populations size in the laboratory experiments). The *in-silico* populations were then constrained to a constant population size of 3,000 and experienced random sexual reproduction for 100 generations with no mutation. Inheritance was considered fully independent among loci. Just before implementing the bottleneck events, each population was duplicated. One of the duplicates was kept as the control population, and continued to experience random sexual reproduction until sampling occurred. The other duplicated population was submitted to three bottleneck events in a row, of identical magnitude. After each bottleneck, the population recovered its initial size in a single generation. After the last bottleneck event, each population was allowed to reproduce for 30 generations before sampling. At the last generation, just before sampling, reproduction of parent individuals generated 81000 individuals (i.e., the mean census size of the real populations), in both the control and bottlenecked populations. Then, 30 virtual individuals were sampled and their genotypes recorded. Two such *in-silico* simulations were conducted 100 times each, one with a population size reduction (bottleneck strength) of 1/333 and another with a population size reduction of 1/1000. Analyses of these *in-silico* generated data were performed with Bottleneck exactly as described for the laboratory-generated data.

## Results

All replicate data sets generated in our laboratory experiments are available as supporting information (Text S1). Census size (N) estimates of all populations varied between 77,800±21,400 and 96,000±14,200 individuals per population. Measurements of the census size in 5 populations over 10 generations are given as supporting information (Table S1). Effective size (N_e_) estimated with the program MN_e_ for 2 populations allowed us to compute a Ne/N ratio of 1/27 and 1/33.

All measures of genetic variation (gene diversity, F_ST_, and Garza-Williamson index) are given as supporting information (Tables S2, S3, S4 and S5). All F_ST_ values between populations after the migration experiments are >0.3, except for the comparison between SB146 and PB206 (m = 0.05; see figure 1) that yields values between 0 and 0.0758, depending on the replicate considered. Note that Faubet et al. [11] have shown that BayesAss+ estimates optimal migration rates when F_ST_ values are >0.05, when assumptions of the implemented model are not violated in the real demographic history. F_ST_ values must even be higher (>0.1) when assumptions are violated. In most cases, the level of differentiation among the laboratory-cultured populations was thus appropriate for estimation of migration rates with the program BayesAss+.

**Figure 3:**
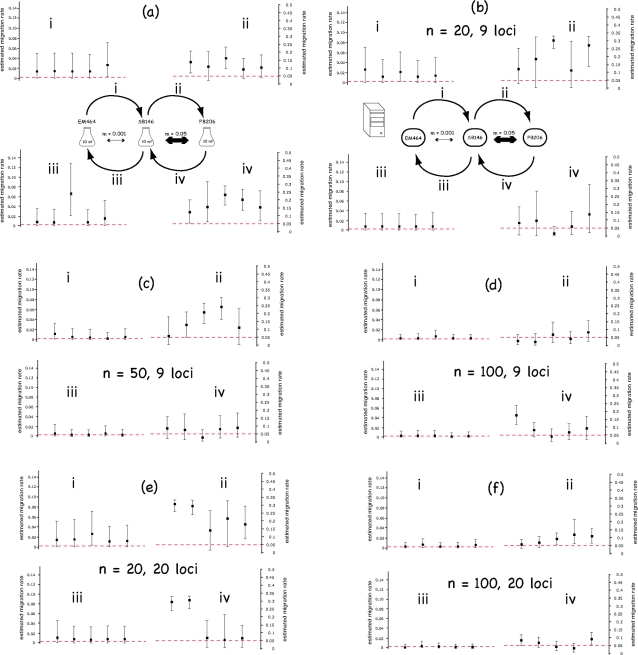
Results from the first migration experiment. Results from the analysis of the in-vitro (a) and *in-silico* (b–f) simulated microsatellite data for the first migration scenario. For each of the four sets of data (a–f), a graph is given for each of the four estimated migration rates (*i*–*iv*). The red dotted line shows the value of the real migration rate implemented. Migration rate estimates (one per replicate) are given together with the confidence interval provided by BayesAss+. Note that the scales on the left and right graphs are different.

**Figure 4:**
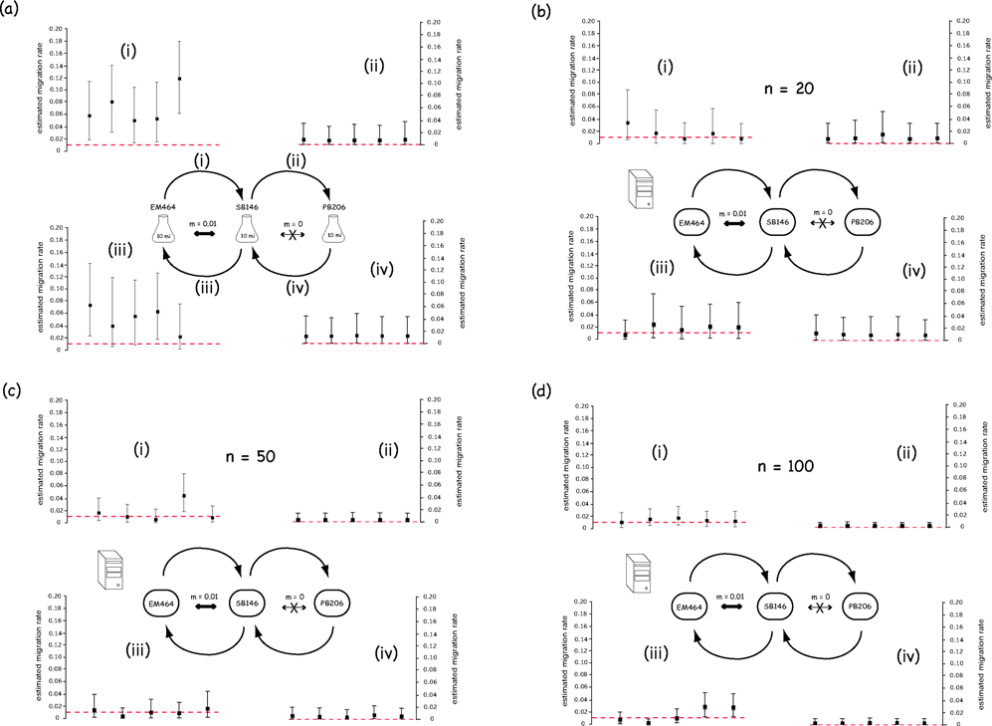
Results from the second migration experiment. Results from the analysis of the *in-vitro* (a) and *in-silico* (b–d) simulated microsatellite data for the second migration scenario. For each of the four sets of data (a–d), a graph is given for each of the four estimated migration rates (*i*–*iv*). The red dotted line shows the value of the real migration rate implemented. Migration rate estimates (one per replicate) are given together with the confidence interval provided by BayesAss+.

BayesAss+ 1.3 estimates of migration rates among laboratory-cultured populations are shown in Figure 3a and 4a, along with 95% confidence intervals. Although the real migration rate values are often (but not always) included in the confidence interval, BayessAss+ systematically overestimates the migration rates implemented in the laboratory experiments. This apparent systematic bias could be due to four different causes: (i) the method intrinsically overestimates migration rates, as far as the conditions of our experiments are concerned, (ii) the implemented method is not biased but the program contains errors, (iii) the population evolution model used by the analytical method is not valid, and (iv) the ratio between effective number and census number of migrating individuals was larger than the ratio N_e_/N. If any of the first two hypotheses was correct, we should observe the same bias with *in-silico* simulated data. Given the large confidence intervals generated by BayesAss+ with the laboratory-cultured data sets, we generated *in-silico* simulated data sets both under conditions similar to our *in-vitro* experimental design (see Methods) and with an increasing number of sampled individuals and/or number of loci. Migration rates estimates from the *in-silico* simulated data sets under conditions similar to the laboratory experiments are shown in Figure 3b and 4b. The systematic over-estimation of migration rates is less pronounced (and sometimes disappear) in the *in-silico* experiments, possibly because of differences between the real evolution process and the models used *in-silico* (for simulations and analysis of data). Figures 3c-f, 4c-d indicate that increasing the number of sampled individuals reduces more drastically the variance of the migration rate estimates than does the number of loci analyzed. Comparison between the long-term migration rates estimates and the BayesAss+ estimates, for one replicate data set, is shown in Table 1.

**Table 1:** migration rates estimated, using three different methods, from replicate 1 data set of the first migration experiment.

	Method	Estimate	Confidence interval
i (m = 0.001)	Lamarc	0.00041	[0.00029–0.00055]
	F_ST_-based	0.00009	N/A
	BayesAss+	0.01377	[0.00038–0.04927]
ii (m = 0.05)	Lamarc	0.00416	[0.00382–0.00454]
	F_ST_-based	0.00182	N/A
	BayesAss+	0.13561	[0.06751–0.20626]
iii (m = 0.001)	Lamarc	0.00268	[0.00189–0.00359]
	F_ST_-based	0.00009	N/A
	BayesAss+	0.00809	[0.00009–0.03562]
iv (m = 0.05)	Lamarc	0.00222	[0.00201–0.00242]
	F_ST_-based	0.00182	N/A
	BayesAss+	0.11989	[0.05193–0.19789]

Results from the Bottleneck analyses are presented in Table 2. In two replicate experiments out of five, the control population, despite that it did not experience any bottleneck, displayed a significant heterozygosity excess. We interpret this as the result of the violation of one of the key assumption of the method, namely that the populations should be in a state of equilibrium between mutation and genetic drift (see Material and Methods). The two experiments of bottleneck generated a significant heterozygosity excess in 4 of the 5 replicates, both when N_e_ was reduced to 9 and to 3 (see Methods). When focusing only on the replicates for which the control data set did not display a significant heterozygosity excess (i.e., replicates 1, 4, and 5), only 4 out of 6 bottleneck events were detected. In the *in-silico* experiments, a large portion of the control populations (51% in the N_e_ = 3 bottleneck experiments and 52% in the N_e_ = 9 bottleneck experiments) displayed a significant heterozygosity excess, even though a bottleneck event was not implemented. These proportions were increased respectively to 65% and 90% in the case of the bottlenecked populations. When no significant heterozygosity excess was detected in the control population (i.e., when the control population does not seem to significantly depart from a mutation-drift equilibrium), a significant heterozygosity excess was detected in 59% and 92% of the corresponding bottlenecked populations. The *in-vitro* results are thus compatible with the *in-silico* observations.

**Table 2:** one-tail p-values associated to the significance of heterozygosity excess calculated with a Wilcoxon sign-rank test (program Bottleneck).

	rep 1	rep 2	rep 3	rep 4	rep 5
control	0.138	**0.003** *	**0.012** *	0.278	0.08
bottleneck 1 (Ne = 9)	**0.007** *	**0.012** *	**0.001** *	0.348	**0.005** *
bottleneck 2 (Ne = 3)	0.097	**0.001** *	**0.012** *	**0.05** *	**0.007** *

* Significant p-values (5% level)

## Discussion

Our study demonstrates the feasibility of the approach that consists in generating molecular genetic data sets by controlling the evolution of laboratory-reared nematode populations, for the purpose of validating methods inferring population history. As noted in the Introduction, *in-vitro* simulations are complementary to *in-silico* simulations and to the analysis of empirical data, and offers the possibility to control and replicate imposed scenarios of population history while avoiding some of the simplifying assumptions implemented in *in-silico* simulations. The major disadvantage of *in-vitro* simulations is that they are much more time-consuming and costly than *in-silico* simulations. Nonetheless, any data set generated *in-vitro* can be repeatedly used to validate inference methods. It is important to realize however that nematode populations cultured in liquid medium exhibit features that can be highly unrealistic in comparison to natural populations of non-model species. For example, shaking of the culture medium generates random meeting, if not random mating, of individuals. Also, the level of control the experimenter has on the demographic parameters is lower than in *in-silico* simulations. For example, although the effective population size can be estimated, and somewhat controlled, fluctuation of this parameter do occur in a laboratory population, the magnitude of this fluctuation depending on how precisely the experimental variables (temperature, culture volume, culture medium,…) are controlled.

Four software applications implementing methods for estimating migration rates among populations are available: Migrate [8], Lamarc [32], IM [9] and BayesAss+ [7]. The three former programs measure long-term migration rates: they assume a constant migration rate over a long period of time (i.e., the time since the two populations separated from a common ancestral population for IM and the time necessary for all alleles to coalesce into a most recent common ancestor for Migrate or Lamarc). Given the small number of generations that can reasonably be used in a laboratory experiment, it seemed not appropriate to evaluate these three programs with our experiments. On the other hand, BayesAss+ is specifically designed to measure recent migration events. It implements a Bayesian method using Markov chain Monte Carlo (MCMC) techniques to estimate posterior probabilities of several population parameters, including migration rates. It assumes that the source populations of immigrants have been sampled (which is clearly the case in our experimental setup) and does not require that the studied populations are in Hardy-Weinberg equilibrium. It is important to emphasize that this method represents a major progress in estimating recent migration rates, and should be preferred over the classical practice of estimating migration from differentiation levels measured among populations (e.g., [33]) that requires populations to be in migration-drift equilibrium, a state probably seldom encountered in natural populations. Evaluation of the performances and limitations of this method and the identification of the conditions under which it works best has been performed previously by Faubet et al. [11] using *in-silico* simulations. Here, we additionally generated and analyzed *in-vitro* simulated datasets for testing the performances of BayesAss+. All migration rates inferred by the program were over-estimates, although these were often included in the confidence interval. Furthermore, our analyses of data sets simulated *in silico* using a model mimicking the laboratory experiments, produced less biased estimates of the migration rates. Therefore, the observed bias could be due to *(i)* unrealistic assumptions (used by the inference method) that might be more violated under *in-vitro* simulations than *in-silico* simulation, or *(ii)* to a systematic, unidentified, error in our implementation of the migration among populations in the laboratory. The latter hypothesis is unlikely because migration implementation was relatively straightforward: homogenization of the culture followed by the transfer of a precise volume of liquid culture from one flask to another. However, our experimental set up uses the assumption that the reproductive success of migrant individuals is identical to that of non-migrant individuals. If this assumption is incorrect, the migration rates inferred by BayesAss+ might actually constitute proper estimates whereas the assumed migration rates would have been under-estimated. If confirmed, this result would suggest that migrant individuals have a higher reproductive success than non-migrant individuals, which is compatible with the findings of a recent study [34] that suggest the potential for strong inbreeding depression occurring in inbred populations of this nematode species.

Conversely, the hypothesis that the program BayesAss+ may, at least in the conditions investigated here, overestimate migration rates is partly supported by the observation that the migration rates are also over-estimated with *in-silico* generated datasets (see for example Figure 3b(i) and 3b(ii)). Note that overestimation at high migration rates could be explained by the low differentiation between populations after 11 generations of high migration. Indeed, as noted above, it was shown by Faubet et al. [11] that BayesAss+ provides best estimates when the F_ST_ characterizing the two populations is >0.05, which was not the case in all our experiments for the two populations connected by the highest migration rate. Although low migration rates were also systematically overestimated, the real values was always included in the confidence intervals. Importantly, this bias tends to disappear when the number of sampled individuals rises to 100, for both low and high migration rates.

An important result of our *in-silico* simulations analyses (Figures 3 and 4) is that increasing the number of sampled individuals (from 20 to 100) decreases much more dramatically the confidence intervals than does an increasing number of typed loci (from 9 to 20).

As previously mentioned, several programs infer long-term migration rates from molecular data, i.e., they assume that migration rates have remained constant over a long period of time. Although this was not the case in our experiments, we nonetheless estimated long-term migration rates from one replicate empirical data set (1) using Lamarc and (2) assuming an island model of migration (see Methods). This was done to investigate to what extent these methods can generate wrong estimates when their basic assumptions are not met. Table 1 shows these estimates along the ones generated by BayesAss+. The Lamarc and F_ST_-based estimates, that assume a long-term stationary migration scenario, are most of the time very different from the true migration rates. In addition, Lamarc estimates are characterized by a much smaller confidence interval, that never includes the true value. Although the BayesAss+ estimates are also far from the true values (at least in the laboratory experiments, in which n = 20), the corresponding confidence intervals are much larger, reflecting better the uncertainty associated to these estimates. Moreover, in almost three cases out of four, the true migration rate is included within the confidence interval provided by BayesAss+.

Whereas our analyses demonstrate the efficiency of BayesAss+ for the inference of recent migration rates when the number of loci and sampled genotypes per population is sufficiently high, evaluation of the performances of the program Bottleneck was less straightforward. While several methods are designed to estimate the effective size of a population or its fluctuation over time, the program Bottleneck is specifically aimed at detecting a recent bottleneck (within the past 2N_e_-4N_e_ generations). As such, it should detect, from our microsatellite data, the bottlenecks we simulated *in-vitro*. The method is based on the observation that, in a bottlenecked population, rare alleles have a higher probability of being lost than more frequent alleles, such that heterozygosity (or gene diversity [25]) becomes higher than would be expected in an equilibrium population. Hence, using a specific mutation model, the method tests for an heterozygosity excess in the real data set in comparison with an equivalent population in equilibrium conditions [4]. One major assumption from the methods implemented in Bottleneck is that the population has reached, before the bottleneck event, a state of equilibrium (in terms of allele frequencies) between mutation and genetic drift. It is very unlikely, however, that the large populations (see estimated effective size) that we generated in the laboratory by mixing several strains of *C. remanei* (for increasing allelic diversity) reached such an equilibrium. Waiting for this equilibrium to be reached is not practical, because it requires a large number of generations (several N_e_ generations). While it is clear that a major assumption of the method was not met by our data, we did analyze the microsatellite data with the program Bottleneck, in order to test the robustness of the method to violation of one of its key assumption. In the replicates for which the pre-bottleneck laboratory-cultured populations did not significantly depart from a mutation/drift equilibrium (i.e., when the control population did not exhibit a significant gene diversity excess; see replicates 1, 4 and 5 in Table 2), a bottleneck event was detected by the inference program in two replicates out of three, for both levels of population reduction (N_e_ = 9 and N_e_ = 3). Note that, in replicate 5, the control population was not far from a significant gene diversity excess such that the effect of the bottleneck event was probably artificially exacerbated. The in-vitro generated data sets were fully compatible with the data sets generated by computer simulations of the laboratory experiments. First, a high proportion (>50%) of the control populations, for which the size was kept constant over the entire experiment, were erroneously identified as bottlenecked populations. We believe this is explained by the violation of one of the key assumption of the method (i.e., absence of a mutation-drift equilibrium), and is entirely attributable to our experimental setup. Second, in the cases where the control populations were not identified as bottlenecked, the corresponding bottlenecked population was identified as such only in 65% (N_e_ = 9) and 90% (N_e_ = 3) of the cases. Given that each bottleneck event was generated, for each replicate, three times in a row, the test appears to be rather conservative. We therefore conclude that the power of detection by the program Bottleneck is low for bottleneck events of nature and intensity implemented in our *in-vitro* simulations.

## Supporting Information

Text S1 Data sets. Molecular data sets generated through the laboratory-controlled evolution of populations of Caenorhabditis remanei. Each text file is formatted to be read by the program it was analyzed with in this study (BayesAss+ or Bottleneck).
(0.04 MB ZIP)

Table S1 Estimation of the census size over several generations. Estimation of the census size in 5 populations over 10 generations. Each estimate was done by counting the number of individuals present in 5 samples of 10 µl of homogenized liquid culture under a dissecting microscope.
(0.01 MB PDF)

Table S2 Average gene diversity (i.e., expected heterozygosity) of each population in the migration experiments
(0.01 MB PDF)

Table S3 F_ST_ among population pairs in migration experiments
(0.01 MB PDF)

Table S4 Average gene diversity (i.e., expected heterozygosity) of each population in the Bottleneck experiments.
(0.01 MB PDF)

Table S5 Garza-Williamson index for each population of the Bottleneck experiment.
(0.01 MB PDF)
