# Supplementary material for: Controlling Population Evolution in the Laboratory to Evaluate Methods of Historical Inference
Source: PLoS One. 2008 Aug 13;3(8):e2960. doi: 10.1371/journal.pone.0002960 (PMC2491900; doi:10.1371/journal.pone.0002960)
Supplement: Table S1 — Estimation of the census size over several generations. Estimation of the census size in 5 populations over 10 generations. Each estimate was done by counting the number of individuals present in 5 samples of 10 µl of homogenized liquid culture under a dissecting microscope. (0.01 MB PDF) [file pone.0002960.s002.pdf]

Estimation of the census size over several generations

	Population 1	Population 2	Population 3	Population 4	Population 5
Generation 0	86800 ± 15200	88200 ± 15400	77800 ± 21400	86800 ± 9100	84300 ± 11800
Generation 5	80000 ± 8000	84500 ± 13200	85300 ± 15700	96000 ± 14200	89600 ± 7100
Generation 10	91000± 13400	86000 ± 9900	88600 ±14400	89800 ± 18300	79500 ± 7800
