# Supplementary material for: Controlling Population Evolution in the Laboratory to Evaluate Methods of Historical Inference
Source: PLoS One. 2008 Aug 13;3(8):e2960. doi: 10.1371/journal.pone.0002960 (PMC2491900; doi:10.1371/journal.pone.0002960)
Supplement: Table S2 — Average gene diversity (i.e., expected heterozygosity) of each population in the migration experiments (0.01 MB PDF) [file pone.0002960.s003.pdf]

Average gene diversity (i.e., expected heterozygosity) of each population in the migration experiments

	Migration experiment 1 (m = 0.001/0.05)			Migration experiment 2 (m = 0/0.01)		
	Pop EM464	Pop SB146	Pop PB206	Pop EM464	Pop SB146	Pop PB206
replicate 1	0.298	0.423	0.582	0.109	0.683	0.626
replicate 2	0.305	0.589	0.599	0.012	0.609	0.789
replicate 3	0.242	0.389	0.534	0.215	0.669	0.703
replicate 4	0.293	0.506	0.516	0.030	0.662	0.608
replicate 5	0.258	0.546	0.639	0.118	0.632	0.707
