# Supplementary material for: Controlling Population Evolution in the Laboratory to Evaluate Methods of Historical Inference
Source: PLoS One. 2008 Aug 13;3(8):e2960. doi: 10.1371/journal.pone.0002960 (PMC2491900; doi:10.1371/journal.pone.0002960)
Supplement: Table S3 — FST among population pairs in migration experiments (0.01 MB PDF) [file pone.0002960.s004.pdf]

$F_{ST}$ among population pairs in migration experiments

| | Migration experiment 1 (m = 0.001/0.05) | | Migration experiment 2 (m = 0/0.01) | |
|---|---|---|---|---|
| | EM464-SB146 | SB146-PB206 | EM464-SB146 | SB146-PB206 |
| replicate 1 | 0.579 | 0.044 | 0.521 | 0.433 |
| replicate 2 | 0.608 | 0.017 | 0.355 | 0.334 |
| replicate 3 | 0.459 | -0.020 | 0.546 | 0.431 |
| replicate 4 | 0.582 | 0.075 | 0.457 | 0.441 |
| replicate 5 | 0.555 | 0.029 | 0.314 | 0.341 |
