# Supplementary material for: Controlling Population Evolution in the Laboratory to Evaluate Methods of Historical Inference
Source: PLoS One. 2008 Aug 13;3(8):e2960. doi: 10.1371/journal.pone.0002960 (PMC2491900; doi:10.1371/journal.pone.0002960)
Supplement: Table S4 — Average gene diversity (i.e., expected heterozygosity) of each population in the Bottleneck experiments. (0.01 MB PDF) [file pone.0002960.s005.pdf]

Average gene diversity (i.e., expected heterozygosity) of each population in the Bottleneck experiments

	control +	bottleneck (Ne=9)	bottleneck (Ne=3)
replicate 1	0.719	0.773	0.623
replicate 2	0.606	0.644	0.662
replicate 3	0.611	0.642	0.595
replicate 4	0.679	0.635	0.674
replicate 5	0.636	0.675	0.646
