# Supplementary material for: Controlling Population Evolution in the Laboratory to Evaluate Methods of Historical Inference
Source: PLoS One. 2008 Aug 13;3(8):e2960. doi: 10.1371/journal.pone.0002960 (PMC2491900; doi:10.1371/journal.pone.0002960)
Supplement: Table S5 — Garza-Williamson index for each population of the Bottleneck experiment. (0.01 MB PDF) [file pone.0002960.s006.pdf]

Garza-Williamson index for each population of the Bottleneck experiment

	control +	bottleneck (Ne=9)	bottleneck (Ne=3)
replicate 1	0.265	0.245	0.256
replicate 2	0.248	0.228	0.301
replicate 3	0.258	0.286	0.294
replicate 4	0.257	0.281	0.287
replicate 5	0.298	0.220	0.266
